# Evaluation of a mentorship matchmaking event at an academic research institution to reinforce the scientific workforce pathway for underrepresented minority groups

**DOI:** 10.1186/s12909-024-06410-1

**Published:** 2025-01-20

**Authors:** Sirena Gutierrez, Jennifer A. Seuferer, Angel-Max Guerrero, Yazmin Carrasco, Kirsten Bibbins-Domingo, Tung Nguyen, Leticia Márquez-Magaña, Todd Nystul, Meghan D. Morris

**Affiliations:** 1 Department of Epidemiology and Biostatistics, University of California, San Francisco, CA USA; 2 Helen Diller Family Comprehensive Cancer Center, Office of Education & Training, University of California, San Francisco, CA USA; 3 Center for Science Education and Outreach, University of California, San Francisco, CA USA; 4 Graduate School of Medical Sciences, Weill Cornell Medicine, New York, NY USA; 5 Department of Biology, San Francisco State University, San Francisco, CA USA

**Keywords:** Diversity, Mentorship, Research institutions, Higher education

## Abstract

**Background:**

Mentorship and research experiences are crucial for STEMM career entry and advancement. However, systemic barriers have excluded people from historically underrepresented groups.

**Methods:**

In 2021, a virtual “matchmaking event” was held to connect NIH-funded research mentors with historically underrepresented trainees and initiate mentored research experiences. Survey data collected over 12 months was analyzed to evaluate the program’s success considering the number of mentor-trainee connections, mentor-trainee research experience matches, and NIH diversity supplement application status. Statistical tests, including student’s t-test, ANCOVAs, and chi-square tests, evaluated differences between attendee groups and survey time points.

**Results:**

Out of 314 mentors contacted and 99 registered trainees, 113 mentors and 92 trainees participated. Among mentors (*n* = 73), 53% identified as women, 56% as non-Hispanic white, and a majority (81%) reported being the first in their family to attend college. Among trainees (*n* = 79), about two-thirds (67%) identified as women, 47% identified as Hispanic/Latinx, and 15% identified as Black/African American. Both mentors and trainees were extremely satisfied with the overall event (57% and 69%, respectively) and would recommend it to others (74% and 90%, respectively). Most mentor participants established at least one mentor-trainee connection after the event (*n* = 64, 57%), a mentor-trainee research experience match (*n* = 40, 35%), and planned to submit an NIH diversity supplement (*n* = 31, 27%). Many trainees obtained paid positions through the mentor-trainee research experience. One year after the event, 11 trainees secured NIH diversity supplement funding with their mentors.

**Conclusions:**

The matchmaking event began bridging a much-needed gap in the research pathway by creating opportunities for trainees to connect with mentors and obtain funded research opportunities.

**Supplementary Information:**

The online version contains supplementary material available at 10.1186/s12909-024-06410-1.

## Background

Racial and ethnic minorities, women, and persons with disabilities, hereafter referred to as historically underrepresented (HU) groups, have faced exclusion and barriers in various long-standing and ingrained systems—for example, political, legal, economic, health care, education systems—that have limited their participation and representation in higher academic institutions due to discriminatory practices [1]. Specifically, historical and ongoing patterns of discrimination and oppression, operating through biases, policies, and overt practices, continue to have a cumulative effect on hindering the inclusion of HU groups over time. Despite legislative and social policies such as the Civil Rights Act of 1964, Title IX, and the Americans with Disabilities Act of 1990 to remove barriers, HU groups continue to be underrepresented in higher education and science, technology, engineering, math, and medicine (STEMM) careers [2]. Among HU groups, Black, American Indian, and Hispanic adults are less likely to earn degrees in STEMM than in other degree fields, and they continue to make up a lower share of STEMM bachelor’s degree recipients relative to their share of the adult population. In 2019, the proportion of STEMM bachelor’s degree recipients compared to the adult population aged 20 to 34 years was 9% vs. 14% for Black, 0.4% vs 1% for American Indians, and 16% vs. 21% for Hispanic [3]. Black and Hispanic workers also remain underrepresented in the STEMM workforce compared with their share of the nation’s workforce (9% vs. 11% for Black workers and 8% vs. 17% for Hispanic workers), including in computing jobs, a field that has had considerable growth in recent years [4].

### The value of diversity

Expanding representation in the scientific enterprise enhances innovation and scientific rigor by bringing together different perspectives to scientific approaches [5–7]. Racial diversity in the workforce has been associated with increased innovation and critical thinking across various social and professional settings [8, 9]. This may be partly driven by a broader set of lived experiences that provide a more comprehensive range of social, linguistic, navigational capital and other forms of cultural wealth [10]. Likewise, HU students may be more likely to ask new scientific questions that expand perspectives to drive a broader research agenda [11, 12]. They are also more likely to focus attention on understudied health issues that affect a diverse set of communities [13, 14]. Diversifying the workforce reinforces a cycle that propels and sustains further diversification [11]. As a result, expanding representation in the scientific enterprise enhances innovation and scientific rigor by bringing together different perspectives to scientific approaches [5–7].

### Barriers to increasing diversity in STEMM

Barriers to increasing diversity in STEMM exist along the continuum of recruitment, retention, and achievement of HU students in higher education. HU STEMM majors disproportionately face historical and institutional barriers to success, including high financial need, social and cultural isolation, lack of science capital, non-inclusive or hostile campus climates, as well as higher rates of stereotype threat, discrimination, and bias in and outside of the classroom [15, 16]. In addition, research suggests that the invitation by scientists to participate in a research experience is limited by gender, racial, and ethnic biases against HU students in STEMM [17]. Specifically, a field experiment found that faculty were more than twice as likely to respond to mentorship email requests from White male students compared to other racial groups and women. The bias against minority racial groups and women was further exacerbated in higher-paid disciplines. This can lead to self-imposed psychological barriers, resulting in imposter syndrome and reduced confidence in pursuing STEMM careers. Similar accounts of discriminatory hiring and promotion practices, hostile environments, and wage gaps are pervasive in the job market, hindering the retention and ability of HU individuals to succeed in the STEMM workforce [4, 18–20].

Furthermore, the intersectionality framework suggests that multiple forms of inequality and disadvantage may compound themselves in people who identify with several historically underrepresented identities [18]. Barriers to STEMM education and workforce participation are further shaped by how these intersecting identities lead to differential opportunities and challenges. The central emphasis of this study was on racial and ethnic underrepresentation in the sciences. However, it is necessary to acknowledge that addressing the multidimensional aspects of social identity (e.g., race, gender, nativity, disability) used to disadvantage individuals is crucial for targeted research interventions to increase HU individuals in STEMM.

### Benefits of mentored research experiences

Many programs that have attempted to improve the representation of HU students in STEMM higher education and STEMM careers by increasing the number of HUs enrolled in undergraduate STEMM majors have had only modest success [21, 22]. However, success has been improved in programs that offer opportunities to apply classroom education to mentored research experiences that provide important experiential knowledge, professional connections, and enhanced scientific identity [23–27]. These experiences support the pursuit of careers in research by providing the skills and knowledge necessary for success, promoting a sense of science identity at a critical early point in the career, and increasing awareness about STEMM career paths [27–30]. Indeed, surveys of students who participate in undergraduate research experiences report that these experiences increased their commitment to science in general, created awareness of previously unknown areas of science, and enhanced understanding and appreciation of the research process [28, 29, 31]. Research experiences lasting at least two years were positively correlated with graduate school success [30]. Notably, mentored research experiences at the undergraduate and postbaccalaureate level have been found to not only prepare students for graduate school but also lay a foundation for entry into other types of STEMM careers that may not require a PhD, such as in industry or policy [32].

### Aims

As part of a university’s diversity, inclusion, and equity program, an event aimed at bringing together research mentors from a research-intensive university with current HU undergraduate students and those who had recently completed their bachelor’s degree from other local universities was developed, specifically, with a central emphasis on increasing racial diversity in the STEMM sciences. The half-day virtual “matchmaking event” (MME) was designed to spark mentor-trainee connections, resulting in mentored research experiences. Survey data collected from MME attendees across a 12-month period was analyzed to evaluate program satisfaction and impact. The overall goal of the matchmaking event is to provide scholars who are looking for postbaccalaureate research opportunities and meet the suggested eligibility criteria for funding through the NIH Research Supplements to Promote Diversity in Health-Related Research (“Diversity Supplements”) to meet with UCSF faculty who are looking to hire research technicians.

## Methods

### Participant recruitment

The target audience was current and recent undergraduate students who met the suggested eligibility criteria for NIH diversity supplement applications [33], with an emphasis on individuals from historically underrepresented racial and ethnic groups in STEMM. The event was advertised using emails to select degree programs at local universities and colleges with a large HU student population, social media, and advertisements at STEMM research meetings starting in October 2020. Trainees were recruited from a wide range of universities, including California State Universities, schools within the University of California system, and other private institutions. For a comprehensive list, see Supplementary Appendix 1. Research mentors from the University of California San Francisco (UCSF) were recruited using a combination of active outreach and passive advertisement starting in November 2020, two months before the event. Emails were sent to the university’s list of departmental diversity leaders and NIH-funded research faculty and staff. Additionally, mentors from HU groups, as well as those with prior experience mentoring HU students, were purposely invited. Although many mentors did have NIH funding eligible to support diversity supplements, this was not a formal criterion for mentor participation. The mentor pool included participants at various career stages, including staff scientists and postdoctoral fellows. The event was advertised in campus newsletters. Periodic emails advertising the matchmaking event were sent to both mentors and trainees. This pilot evaluation study will help inform power analysis for subsequent evaluations of this annual event.

### Matching

The matching process occurred in several stages. First, when registering, faculty and trainees provide their top 3 research areas of interest. After registration, students are screened to confirm they meet the requirements for an NIH diversity supplement. A couple weeks before the event, after registration closes, faculty and trainees are sent 1) a list of all trainee and faculty registrants and 2) a survey to choose their top 5 people with which to interview. This information is then used in a customized algorithm (provided upon request) that matches faculty and students based on their interview choices and their areas of research interest.

### The event

The Matchmaking Event (MME) was a 4-h virtual event designed to facilitate mentor-trainee connections between participants. It began with all participants gathering in one Zoom meeting room. The event started with a warm welcome and an introduction by the team. A presentation followed, focused on diversity supplements and the benefits and resources available through the Post-baccalaureate Research Opportunity to Promote Equity in Learning (PROPEL) program. In essence, PROPEL is a post-baccalaureate program that offers a unique combination of a paid research position in a UCSF lab along with career and professional development training sessions, scientific courses, and networking opportunities for HU trainees [34]. MME attendees who were hired into a full-time position at UCSF and expressed strong interest in pursuing PhD or MD/PhD programs were invited to join the PROPEL program for additional training and professional development support. For the institutional context and additional details of the event, see Supplemental Appendix 1.

After the introductory session, mentors and trainees moved into separate pre-arranged Zoom meetings for their 10-min interviews. Both mentors and trainees had received a schedule beforehand to know their interview slots. Each 10-min interview was followed by a 5-min break to allow for notetaking and to let trainees transition to their next interview. This process continued for 2.5 h, with a 30-min break in the middle. Once all the interviews were completed, everyone reconvened in the original Zoom room for a presentation on potential next steps for the participants and a question-and-answer session.

As mentioned above, trainees and mentors were matched for interviews before the day of the MME and were provided short biographies of their matches to help them prepare for their interviews. Before the event, trainees and mentors had completed a survey indicating their preferences for interview partners based on a database created from the registration information. Mentors could also specify their available interview time slots. The pairing for the available interview slots was determined by utilizing this information and considering mentor and trainee research interests. For additional details on the matching protocol, see Supplemental Appendix 2; for examples of the informational interview guides provided to participants prior to the event, see Supplemental Appendix 3.

The primary learning objectives of the event were to enable participants to gain interview and communication skills while discussing their research agenda, skillsets, and interests by participating in up to eight 10-min interviews. Additionally, participants were encouraged to understand the diversity supplement funding mechanism and identify institutional resources offered through PROPEL and other related programs. Overall, the MME contributed to the broader goal of establishing a sustainable infrastructure to foster meaningful inclusion of HU trainees, research staff, and principal investigators at UCSF. The ultimate aim was to make inclusive research a norm within the institution.

### Data collected

**Fig. 1:**
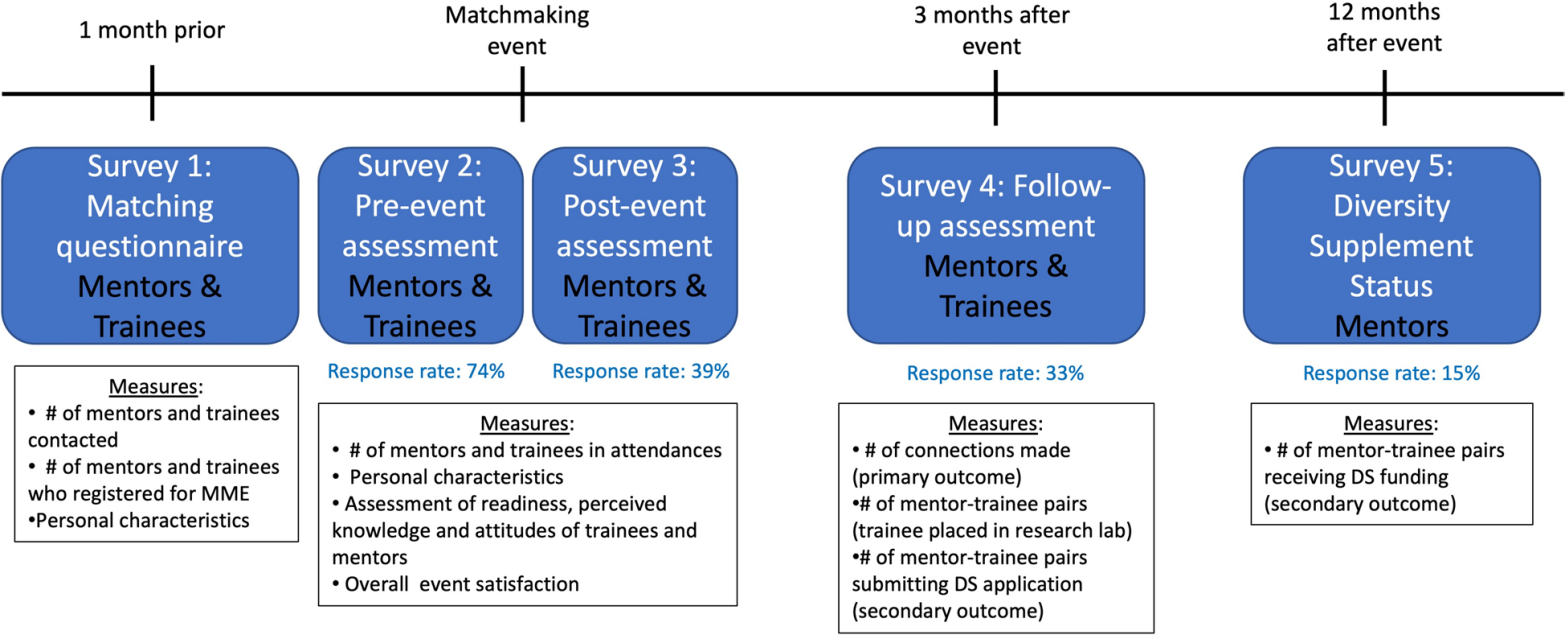
Data sources and evaluation structure of the 2021 matchmaking event

Figure 1 displays the MME evaluation data collection time points. (1) Survey 1: A digital matchmaking database created with SmartSheets (Smartsheets, Inc.) was utilized to collect trainee data on research experience (not required), research interests, future career goals, and demographic information. Additionally, resumes from each trainee were collected. Both trainees and mentors completed an event registration questionnaire. The responses from mentors and trainees were utilized to create the speed interview pairs. The matchmaking database was also made accessible to faculty unable to attend the event, enabling them to search for potential matches independently. (2) Surveys 2 and 3: A pre-event survey (Survey 2) was administered to all in attendance during the first 10 min. This questionnaire gathered information on their professional and training background, sociodemographic characteristics, knowledge of the NIH diversity supplement application process, and assessed the perceived value of diversity and research self-efficacy. For trainees only, it also evaluated their science identity. Immediately following the event, a post-event survey (Survey 3) was administered via email to mentor and trainee attendees. This questionnaire assessed event satisfaction, intentions for follow-up conversations, and overall understanding of the NIH Diversity Supplement application process. (3) Survey 4: Three months after the matchmaking event, information was solicited about post-event mentor-trainee connections reflecting both trainee and mentor reporting an intent to pursue follow-up conversations or a research collaboration (number, placements, and future plans). (4) Survey 5: Twelve months after the event, information from mentors was gathered about sustained mentor-trainee research experiences and the status of NIH Diversity Supplement applications. All questionnaires were administered using Qualtrics, with a unique questionnaire tailored to mentors and a separate version tailored to trainees. Names and email addresses were collected to facilitate participant linking across time points and confirm mentor-trainee pairing. After data linkages were performed to generate a participant ID, the name, email, and internet protocol address identifiers were removed from the database, and only a de-identified version of the entire repository was made available.

### Measures

#### Outcomes

The primary outcome of interest was a binary measure indicating whether a mentor-trainee ‘connection’ was made following the MME. Participants were categorized as having a mentor-trainee connection if a trainee or mentor participant reported having at least one follow-up conversation in survey 4. Secondary outcomes collected during survey 4 included a binary indicator for a mentor-trainee match as defined by a trainee and/or mentor responding that they were currently in discussion about joining or hosting a research group and a mentor’s intention to apply for a diversity supplement to support the trainee as indicated by mentor responses. In survey 5, the secondary outcome was the number of mentor-trainee pairs receiving an NIH-funded diversity supplement.

#### Other variables of interest

Measures of event satisfaction were captured throughout the post-session survey; participants responded to: “Overall, how satisfied are you with the matchmaking event?” “How effective was the speed interviewing session in helping you match with an eligible faculty member?” and “I would recommend this event to a friend or colleague.” Responses were coded along a Likert scale.

We explored several other variables, including science identity, research self-efficacy, and perceived value of diversity, based on prior evidence linking these factors with retention and success in STEMM [35–37]. However, due to limited variability and reduced statistical power, we provide details on their operationalization and descriptive statistics in Supplemental Appendix 4.

### Analysis

Descriptive statistics for attendee characteristics were generated and categorized by mentor and trainee groups. Statistical tests, including student’s t-test, ANCOVAs, and chi-square tests, were employed to identify significant differences among attendee groups and survey time points. To account for potential selection bias, an examination was conducted to identify any significant sociodemographic characteristics among participants who completed surveys over the 12-month period. All *p* values were two-sided; α = 0.05 was considered the cutoff for statistical significance. All statistical analyses were conducted using Stata version 17 (StataCorp LLC, College Station, TX). The University of California, San Francisco institutional review board approved the study and the waiver of informed consent as this study was deemed exempt from human subjects’ research procedures (IRB #21–33387). Clinical trial number: not applicable.

## Results

### Attendee characteristics

Of the 314-mentors emailed, 113 (36%) attended the matchmaking event. Of the 99 trainees who signed up for the matchmaking database, 92 (93%) attended the matchmaking event. Of the 205 total attendees, 152 (95%) completed the pre-session survey; 73 (89%) were mentors and 79 (100%) were trainees. Of the 73 mentors, there were 29 (40%) professors, 21 (29%) associate professors, 12 (16%) assistant professors, and 3 (4%) postdoctoral fellows. Most mentors (53%) identified as women, as non-Hispanic white (56%), and as the first in their families to attend college (81%). Among trainees (*n* = 79), about two-thirds (67%) identified as women and 47% identified as Hispanic/Latinx, and 15% identified as Black/African American. The most cited reason for a mentor's attendance was to ‘identify a mentee to host in their research group’ (80%), followed by ‘to meet trainees interested in research’ (10%). Among trainees, the most frequently cited reason for attendance was ‘to position themselves for a job at UCSF in research’ (42%) and ‘to identify a faculty member and apply for an NIH Diversity Supplement (30%). Most participants reported either having no or little prior knowledge of the NIH diversity supplement mechanism or application process (55% for mentors and 87% for trainees). Tables 1 and 2 provide a full description of mentor and trainee characteristics. Response rates were 74% for the pre-event survey, 39% for the post-event survey, 33% for the first follow-up survey, and 15% for the second follow-up survey, which was administered to faculty only. Non-participation in the subsequent follow-up surveys could be due to participants not making a mentor-trainee pairing; however, earlier surveys assess satisfaction and reasons pairings were not made. During the 524.6 person-months of follow-up (average 3.5, maximum 12 months), there were no significant differences in gender, race/ethnicity, or other key characteristics across the follow-up surveys compared to the pre-session sample (*p* > 0.05).

**Table 1:** Matchmaking event mentor characteristics (*n* = 73)

	No. (%)
Gender	
Female	39 (53.4)
Male	30 (41.1)
Genderqueer or non-conforming	0 (0.0)
Missing	4 (5.5)
Racial and ethnic group^a^	
White	41 (56.2)
African American/ Black	1 (1.4)
Asian^b^	16 (21.9)
Filipino, Hmong, Vietnamese	1 (1.4)
Hispanic/Latinx	5 (6.9)
Other	5 (6.9)
Missing	4 (5.5)
Reported a disability	2 (2.7)
First in family to attend college	59 (80.8)
Main research area of interest	
Bioengineering	6 (8.2)
Cancer biology and cell signaling	6 (8.2)
Computational biology	3 (4.1)
Developmental and stem cell biology	4 (5.5)
Epidemiology and biostatistics	5 (6.9)
Human genetics	4 (5.5)
Immunology	8 (11.0)
Neurobiology	14 (19.2)
Reproductive science	2 (2.7)
Tissue/organ biology and endocrinology	2 (2.7)
Vascular and cardiac biology	2 (2.7)
Virology and microbial pathogenesis	2 (2.7)
Other	12 (16.4)
Missing	3 (4.1)
Main reason for attending matchmaking event	
To identify a mentee/trainee to host in my lab/research group	58 (79.5)
To meet trainees interested in research	7 (9.6)
To meet other faculty at UCSF	0 (0.0)
To learn more about applying for a DS	4 (5.5)
I’m not quite sure but it seemed like a cool event	1 (1.4)
Missing	3 (4.1)
Prior knowledge of DS application process	
None at all	16 (21.9)
A little	24 (32.8)
A moderate amount	15 (20.6)
A lot	10 (13.7)
A great deal	5 (6.9)
Missing	3 (4.1)
Position at UCSF	
Assistant professor	12 (16.4)
Associate professor	21 (28.8)
Full professor	29 (39.7)
Staff scientists	1 (1.4)
Postdoc fellow	3 (4.1)
Other	4 (5.5)
Missing	3 (4.1)

Data obtained by participants who completed the pre-session survey prior to the NIH event

*Abbreviations*: *NIH *National Institute of Health, *DS*, NIH Diversity supplement, *PROPEL *Post-baccalaureate Research Opportunity to Promote Equity in Learning, *SFSU *San Francisco State University, *UCSF *University of California San Francisco

^a^Respondents could select more than one option

^b^Other than Filipino, Hmong, or Vietnamese

**Table 2:** Matchmaking event trainee characteristics (*n* = 79)

	No. (%)
Gender	
Female	53 (67.1)
Male	24 (30.4)
Genderqueer or non-conforming	2 (2.5)
Racial and ethnic group^a^	
White	7 (8.9)
African American/ Black	12 (15.2)
Asian^b^	9 (11.4)
Filipino, Hmong, Vietnamese	8 (10.1)
Hispanic/Latinx	37 (46.8)
Other	6 (7.6)
Reported a disability	10 (12.7)
Main research area of interest	
Bioengineering	2 (2.5)
Cancer biology and cell signaling	5 (6.3)
Computational biology	10 (12.7)
Developmental and stem cell biology	6 (7.6)
Epidemiology and biostatistics	6 (7.6)
Human genetics	3 (3.8)
Immunology	7 (8.9)
Neurobiology	17 (21.5)
Reproductive science	3 (3.8)
Tissue/organ biology and endocrinology	3 (3.8)
Vascular and cardiac biology	1 (1.3)
Virology and microbial pathogenesis	11 (13.9)
Other	4 (5.1)
Missing	1 (1.3)
Main reason for attending matchmaking event	
I’m not quite sure but it seemed like a cool event	2 (2.5)
To position myself for a job at UCSF in research	33 (41.8)
To identify a faculty member and apply for a DS	24 (30.4)
To learn more about research as a career opportunity	15 (19.0)
To meet other students interested in research	0 (0.0)
To meet faculty at UCSF who are conducting research	5 (6.3)
Decision to attend event driven by PROPEL	
Not at all	6 (7.6)
A little	14 (17.7)
A lot	59 (74.7)
Prior knowledge of DS application process	
None at all	24 (30.4)
A little	45 (57.0)
A moderate amount	0 (0.0)
A lot	8 (10.1)
A great deal	2 (2.5)
SFSU is their current institution	21 (27.6)
Current status of trainee	
Undergraduate student	56 (70.9)
Post-baccalaureate student	13 (16.5)
Bachelor’s degree holder, not currently a student^c^	8 (10.1)
Master’s degree holder, not currently a student^c^	2 (2.5)

Data obtained by participants who completed the pre-session survey prior to the NIH event

*Abbreviations*: *NIH *National Institute of Health, *DS *NIH diversity supplement, *PROPEL *Post-baccalaureate Research Opportunity to Promote Equity in Learning, *SFSU *San Francisco State University, *UCSF *University of California San Francisco

^a^Respondents could select more than one option

^b^Other than Filipino, Hmong, or Vietnamese

^c^Indicated highest degree obtained

### Event evaluation metrics

Both mentors (*n* = 35) and trainees (*n* = 45) were extremely satisfied with the overall event (57% and 69%, respectively) and would recommend it to a colleague (94% and 100%, respectively) (*p* > 0.05) (Table 3). However, trainees were more likely to follow up after the event compared to mentors (*p* = 0.02), and mentors knew more about the steps necessary to apply for the NIH diversity supplement (*p* = 0.01). After the event, many participants reported having a high level of knowledge about the NIH diversity supplement mechanism and application process, with 51% of mentors and 40% of trainees indicating they had "a lot" or "a great deal" of knowledge.

**Table 3:** Immediate post-assessment of the NIH diversity supplement matchmaking event, 2021 (*n* = 80)

**Outputs**	Mentors (*n* = 35)		Trainees (*n* = 45)		***p*-value**
	**No**	**%**	**No**	**%**	
Overall satisfaction with the event					0.36
Extremely dissatisfied	1	2.9	0	0.0	
Somewhat dissatisfied	1	2.9	0	0.0	
Somewhat satisfied	13	37.1	14	31.1	
Extremely satisfied	20	57.1	31	68.9	
Would recommend this event to friend/colleague					0.249
Strongly disagree	1	2.9	0	0.0	
Somewhat disagree	1	2.9	0	0.0	
Somewhat agree	7	20.0	5	11.1	
Strongly agree	26	74.3	40	89.9	
Effectiveness of speed interviewing session for forming a match					0.423
Slightly effective	2	5.7	0	0.0	
Moderately effective	10	28.6	12	26.7	
Very effective	14	40	21	46.7	
Extremely effective	9	25.7	12	26.7	
Total number of interviews (range 3–5 +)					0.109
3	1	2.9	3	6.7	
4	9	25.7	4	8.9	
5 or more	25	71.4	38	84.4	
Knowledge on the NIH DS application					0.153
None at all	0	0.0	9	20.0	
A little	4	11.4	18	40.0	
A moderate amount	13	37.1	0	0.0	
A lot	11	31.4	16	35.6	
A great deal	7	20.0	2	4.4	
Pre-event material	n/a	-			n/a
Not useful at all			0	0.0	
Slightly useful			0	0.0	
Moderately useful			5	11.1	
Very useful			20	44.4	
Extremely useful			20	44.4	
Plans to contact at least one interviewee					0.02
No	0	0.0	0	0.0	
Maybe	4	11.4	0	0.0	
Yes	31	88.6	45	100	

Data obtained by participants who completed the post-session survey after the NIH event

*Abbreviations*:* NIH* National Institute of Health, *DS* Diversity supplement

Of the 67 attendees who answered the follow-up survey (Survey 4), 64 (95%) reported having made a mentor-trainee connection after the event (97% and 94% for mentor and trainees, respectively) (Table 4). There were a total of 40 mentor and trainee pairs (referred to as being matched hereafter) established, defined as a participant stating they were talking to a trainee/mentor with the goal of joining their research group. Among these 40 pairs, 31 (78%) reported intending to submit an NIH Diversity Supplement, while 6 (15%) stated they were not; three mentor-trainee pairs did not respond.

**Table 4:** Long-term assessment outcomes from the NIH supplement matchmaking event, from research mentor respondents of survey 4 and 5 (*n* = 67)

	No. or Mean (SD)	%	% based on all mentors who attended the event^*a*^
Post-event participant connections made			
Yes	64	95.5%	56.6%
No	3	5.4%	2.7%
Average number of post-event conversations made by participants	2.4 (1.3)	-	-
*Mentor-trainee match-specific outcomes*			
Currently in discussion about joining/hosting research experience (*n* = 56) ^a^			
Yes	40	71.4%	35.4%
No	12	21.4%	10.6%
Missing	4	7.1%	-
Plan to submit NIH diversity supplement (*n* = 40) ^b^			
Yes	31	77.5%	27.4%
No	6	15.0%	5.3%
Missing	3	7.5%	-
Diversity supplement application status 3-months post event (*n* = 31) ^b^			
Contacting NIH program officer	9	29.0%	8.0%
Preparing proposal	12	38.7%	10.6%
Proposal ready to submit	0	0%	0%
Proposal submitted	10	32.3%	8.8%
Diversity supplement application outcome 12-months post event (*n* = 17)^c^			
No diversity supplement submitted	3	17.7%	2.7%
Currently working on diversity supplement	2	11.8%	1.8%
DS submitted and awaiting outcome	1	5.9%	0.9%
Diversity supplement funded	11	64.7%	9.7%
Diversity supplement submitted but not funded	0	0%	0%

72 participants answered Survey 4, however; we removed 5 participants for not answering any of the outcome items. For mentor-trainee match-specific outcomes there are different sample sizes, given the survey branching logic

Abbreviations: NIH, National Institute of Health, DS, Diversity supplement, SD, Standard deviation

^a^Percentages calculated as a proportion from the total number mentor participants of the MME (denominator: *n* = 113)

^b^The second set of responses in which both mentors and trainees matches answered (*n* = 11) were removed to avoid duplicate response per mentor-trainee match. Trainee response values were used in instances where the research mentor responses were missing data

^c^Survey 5 was only asked among research mentors (reference Fig. 1)

Among trainees who reported joining a research mentor’s group and intending to submit an NIH Diversity Supplement application (*n* = 31), 21 reported they were hired into a paid position, 9 stated they would not be working until NIH Diversity Supplement funding was secured, and none indicated they would be volunteering without pay. Specifically, of the 92 trainee MME participants, 23% reported being hired into paid positions, while 10% indicated they would not be working until NIH Diversity Supplement funding was secured.

Trainees who identified as Filipino, Hmong, or Vietnamese (23%) were more likely to be waiting for NIH Diversity Supplement funding to be secured before working (*p* = 0.027) compared to those who were in paid positions; however, there were no additional significant sociodemographic differences. Among trainees and mentors who matched, mentors were significantly more likely to be White compared to the trainees. However, both groups exhibited similar composition in terms of gender and disability status (Supplemental Table 1). There were no statistically significant sociodemographic characteristics differences between trainees who matched and those who did not (Supplemental Table 2). It is noteworthy that trainees who did not find a match had a higher percentage of women (75% vs. 54%), Asian individuals (18% vs. 0%), and reported having a disability (14% vs. 11%) compared to trainees who did find a match.

### Participant reflections of the event

**Fig. 2:**
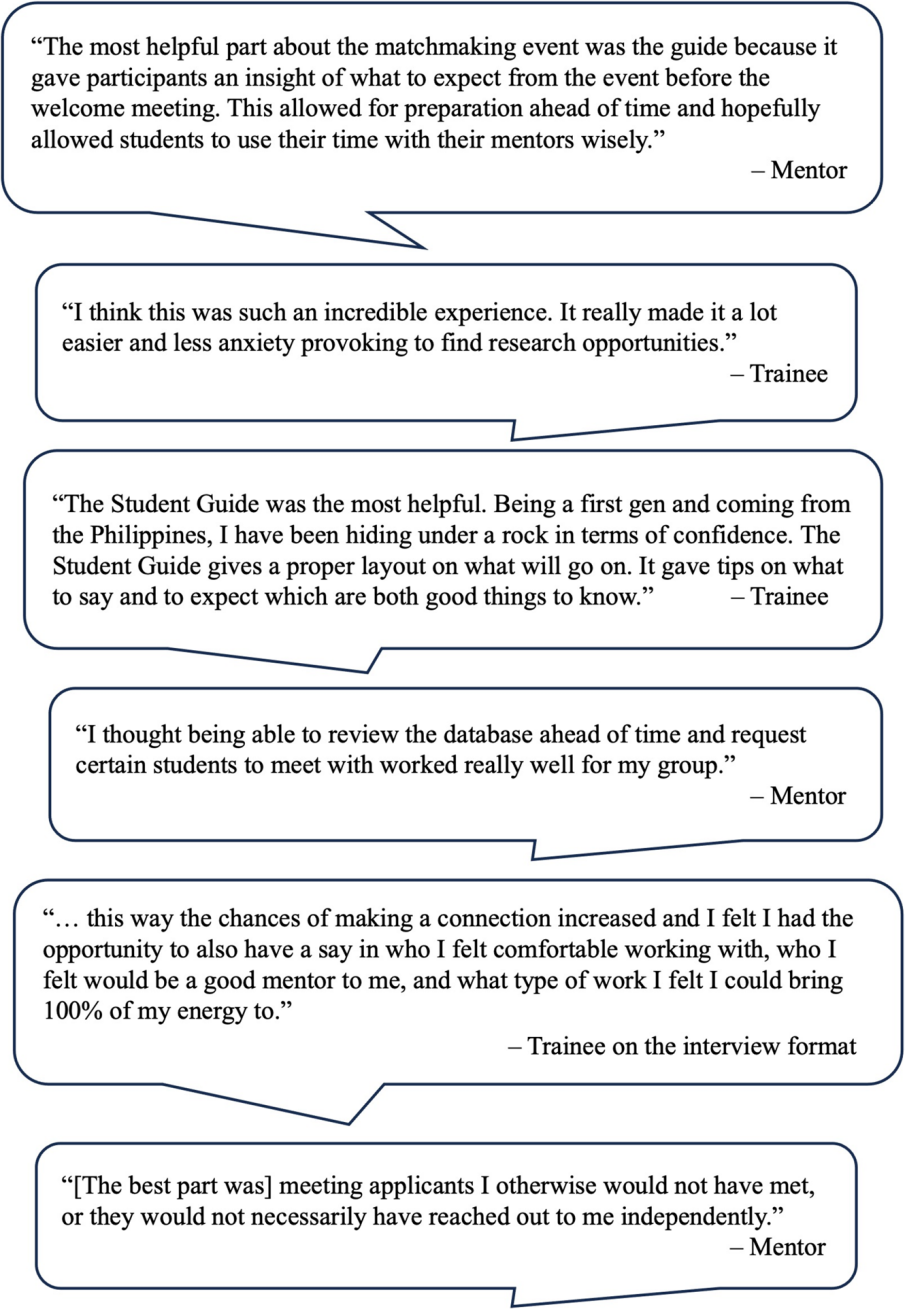
Selected quotes from participants of the MME

Participants also described aspects of the intervention that they found particularly successful toward their goal of developing a mentor-trainee relationship (Fig. 2). Common themes included appreciation for the format, perceived value of the informational guide and database, and overall satisfaction of meeting a diverse group of trainees and mentors.

One trainee shared their experience with the guide:

*Being a first gen and coming from the Philippines, I have been hiding under a rock in terms of confidence. The Student Guide gives a proper layout on what will go on. It gave tips on what to say and to expect.*

Another trainee highlighted the sense of agency they gained from the event:

*I felt I had the opportunity to also have a say in who I felt comfortable working with, who I felt would be a good mentor to me, and what type of work I felt I could bring 100% of my energy to.*

Similarly, a mentor emphasized the benefits of the matching process:

*Being able to review the database ahead of time and request certain students to meet with worked really well for my group.*

### NIH diversity supplement application status

A year after the MME, among those who reported an intention to submit an NIH Diversity Supplement application in survey 4, 17 research mentors provided follow-up information about their application status (*n* = 17, Table 4). Of these, 11 (65%) stated their NIH Diversity Supplement was funded or their notice of award was pending, one (6%) reported the NIH Diversity Supplement was submitted and they were waiting for the outcome, two (12%) were currently working on the NIH Diversity Supplement application, and none stated that their application was submitted and not funded. Although three (18%) stated they did not submit an NIH Diversity Supplement and were no longer working on it, two of these mentors stated they funded the trainee through other mechanisms.

## Discussion

This matchmaking event successfully met our primary goal to facilitate matches that provided scholars from historically underrepresented backgrounds with research experiences in UCSF labs. Most attendees made at least one mentor-trainee connection after the event established a mentor-trainee research experience match. The majority of mentors planned to submit an NIH diversity supplement to support their trainees, and 23% of trainees had been hired into a paid position as part of the mentor-trainee research experience. At one-year post-event, 11 trainees had a funded NIH diversity supplement with the mentor.

Mentor connections and mentored research opportunities during the early career period are crucial for persistence in STEMM [38] but, unfortunately, studies have found that HU trainees typically receive less mentoring than their non-minority peers [39, 40]. Structural factors can lead to HU trainees having differential access to research mentors. Access is often difficult for many reasons, including that trainees may feel intimidated about contacting faculty, trainees may not have knowledge about which faculty have openings in their labs, faculty may not be responsive to the trainees’ attempts at contact, and limits in the trainees’ professional network [41]. Our MME program, therefore, filled a gap for HU scholars who, without the program infrastructure, would have had to independently identify, communicate with, and set up individual meetings with potential mentors. The Matchmaking Event helped address and reduce several barriers by creating new opportunities for trainees to interact with research groups leading to 40 successful matches with research mentors. Notably, of the 31 who reported joining a research position, all (19/19) of those we have follow-up data for remained in the lab for at least 1 year. In addition to achieving this primary goal, the matchmaking event also achieved our secondary goals of increasing awareness of the NIH Diversity Supplement funding mechanism and providing scholars with an opportunity to develop their interview skills. Moreover, the event engaged our faculty and staff in the broader institutional effort to increase diversity, and although we did not measure this in our survey, this likely helped to raise awareness within our community about the importance of diversity in our workforce and foster positive attitudes about these efforts.

Despite the intrinsic rewards, gratification, and experience to grow as researcher mentors, faculty members cite a wide range of barriers, including the institutional context and environment inhibiting engagement in mentoring [16, 42]. Broadly, limited resources, including time, funding, research projects, and administrative and teaching responsibilities, made it challenging for early career investigators to dedicate sufficient time and guidance for mentoring [43, 44]. Additionally, the lack of institutional recognition and incentives for mentoring can discourage faculty from actively engaging in this role. Given that providing need-based financial awards has been shown to increase the persistence and retention of HU populations in STEMM degree programs and careers [45]. Furthermore, limited diversity in faculty members may lead to a lack of understanding of diverse perspectives or experiences, which can impact the mentorship experience of HU trainees [46, 47]. Given the range of positive mentorship outcomes for trainees and mentors alike, research experiences are central to the functioning of higher education institutions and the success of HU students in STEMM [48]. These challenges underscore the need for institutional support and mentoring training programs to help faculty mentor the next generation of investigators. Therefore, when considering the implementation of a similar event in other institutions, we believe the benefits of the Matchmaking Event were augmented by the significant institutional support for the hiring, retention, and training of post-baccalaureate scholars from underrepresented backgrounds.

The benefits of participating in authentic research activities, particularly with a mentor, have been well documented [45, 49–52]. These opportunities have been shown to increase undergraduate students’ graduation rates, enrollment and retention in STEMM graduate programs, science identity, and research self-identify. One example of such activities aimed at promoting mentorship development is the use of speed networking or mentoring events inspired by the speed-dating model [53–55]. These events have been shown to offer benefits such as fostering quick connections and expanding professional networks. However, they also have limitations, such as superficial interactions and limited time for deeper engagement. Similarly, the concept of using information (e.g., databases, questionnaires, rankings) to match mentors and trainees is relatively intuitive and common practice [56]. However, to our knowledge, no event has fully integrated both the pre-matching process and the event itself specifically for HU students in an academic medical center. While previous literature has explored events such as speed mentoring, particularly in conferences and meetings, and database-driven matching, to our knowledge, none have combined these elements in the way we propose. We believe the strength of our approach lies in the combination of recruiting both high-quality trainees and mentors who are genuinely invested, providing relevant information materials before and during the event to ensure participants are prepared for their interviews, and a shared goal (e.g., submitting a diversity supplement application). A common critique across mentor-trainee development programs is that meaningful mentor–mentee relationships require sustained time and commitment [56]. As such, systems and infrastructures (see Supplemental Appendix 1 for details) also need to be in place to create accountability and provide necessary informational and financial support, benefitting both trainees and mentors.

### Limitations

Although our evaluation suggests promising findings in meeting the objectives of the event, we acknowledge that the results may have been underpowered for outcome comparisons across pre-session survey characteristics (i.e., sociodemographic, science belief, efficacy, diversity). As such, estimates examining group differences should be interpreted with caution due to limited statistical power within subgroups. Most of the trainees who participated in the event belonged to racialized minorities or underrepresented gender groups, which limited our ability to draw conclusions for participants from other underrepresented groups.

Another limitation of this study is the adaptation of previously validated scales. While the findings should be viewed within the scope of this exploratory analysis, caution is warranted in generalizing the results to broader populations or asserting new validity for the abbreviated scales used. However, this approach was designed to derive insights relevant to our specific study population (e.g., trainees' training level), as some items were excluded due to their irrelevance to the study’s context, which may have impacted the assessment of the intended constructs.

We also recognize the limitations of using a convenience sample due to resource and time constraints. This sampling method was chosen to allow sufficient time for mentor-trainee pairings and to ensure adequate time for preparing and submitting NIH diversity supplement applications within the fiscal year. Next, while participant quotes were included to enrich our findings, these qualitative insights are limited by the small number of textual comments provided. Future studies should adopt a mixed-methods approach, as this may uncover unrecognized barriers to HU in STEMM and support the development of more comprehensive interventions that may not be captured by a standard quantitative study design.

Lastly, it is important to note that one motivation for attending the MME was the opportunity to join the PROPEL program. This potential overlap could pose a limitation in disentangling the specific long-term effects of attending the MME from those related to participation in the postbaccalaureate research program. Nevertheless, participants may have gained valuable navigational capital, interview skills, and exposure to faculty, regardless of whether they joined a lab or applied for other programs. Additionally, while our study highlights the positive outcomes of the MME, we acknowledge that these benefits were likely enhanced by significant institutional support for the recruitment, retention, and professional development of scholars from underrepresented backgrounds. Therefore, when considering the generalizability and potential implementation of similar events at other institutions, it is essential to account for varying levels of institutional commitment and resources, which may influence the overall impact of such events and their sustainability.

## Conclusions

In summary, the Matchmaking Event facilitated mentor/trainee matches that provided scholars from HU backgrounds with meaningful research experiences, thereby increasing the diversity of the workforce at UCSF. Additionally, the event offered secondary benefits and synergized with other ongoing efforts to promote diversity on campus. We believe our experience can serve as a guide to other institutions as they explore and develop programs to aid mentored research experiences for HU trainees interested in a career in STEMM.

## Supplementary Information

Supplementary Material 1.

## Data Availability

The datasets generated and/or analyzed during the current study are publicly available on GitHub (https://github.com/sirenagtz/MME_Evaluation).

## Abbreviations

HU: Historically underrepresented
JUSTICE: Joining Underrepresented Minorities Students and Trainees with Investigators in Collaboration and Education
MME: Matchmaking event
NIH: National Institutes of Health
PROPEL: Post-baccalaureate Research Opportunity to Promote Equity in Learning
SD: Standard deviation
STEMM: Science, technology, engineering, math, and medicine
UCSF: University of California, San Francisco
