# Supplementary material for: Evaluation of a mentorship matchmaking event at an academic research institution to reinforce the scientific workforce pathway for underrepresented minority groups
Source: BMC Med Educ. 2025 Jan 20;25:95. doi: 10.1186/s12909-024-06410-1 (PMC11744948; doi:10.1186/s12909-024-06410-1)
Supplement: Supplementary file 1 — Supplementary Material 1. [file 12909_2024_6410_MOESM1_ESM.pdf]

Supplementary Information for "Evaluation of a mentorship matchmaking event at an academic research institution to reinforce the scientific workforce pathway for underrepresented minority groups"

Supplemental Appendix 1. Institutional Context and Additional Details of the Matchmaking Event

Supplemental Appendix 2. Details on the Matching Protocol Before the Event

Supplemental Appendix 3. NIH Diversity Supplement Matchmaking Event 2021 Faculty and Student Guides

Supplemental Appendix 4. Pre-Event Survey Measures on the Perceived Benefit of Diversity Supplement, Scientific Identity, Research Self-Efficacy, and Value of Diversity

Supplemental Table 1. Matchmaking Event Attendee Characteristics Among those who Made a Match, by Mentors and Trainees

Supplemental Table 2. Matchmaking Event Attendee Characteristics of Trainees, by Whether they Made a Match

Supplemental Appendix 1. Institutional Context and Additional Details of the Matchmaking Event

Institutional Context

Historical, cultural, and structural barriers have had a significant impact on the representation of HU students pursuing a STEMM career after college including at the academic research institution of the University of California, San Francisco (UCSF). In 2022, 20% of medical students and 24% of graduate school students at UCSF identified as belonging to an HU group (1), compared to 41% of the racial and ethnic minoritized US adult population in 2021 (2). To address this disparity and improve the diversity – and consequently, the effectiveness – of UCSF's research teams, several initiatives have been introduced, including a mentored research experience matchmaking event.

One such initiative was the formation of the Joining Underrepresented Minorities Students and Trainees with Investigators in Collaboration and Education (JUSTICE) working group, in partnership with the UCSF Research Development Office. The group developed resources for faculty, such as a library of successful diversity supplement applications, a website with information about eligibility, application steps, templates, and guides for submitting materials (3). They also collaborated with the UCSF Human Resources Department and the Office of Contracts and Grants to resolve administrative hurdles to submitting grant applications and hiring scholars into positions funded through NIH diversity supplements.

Additionally, existing programs at UCSF with a proven track record in promoting diversity in science, such as SF BUILD (Building Infrastructure Leading to Diversity), the Helen Diller Family Comprehensive Cancer Center's Office of Education and Training, the UCSF Center for Science Education and Outreach, and UCSF PhD programs, played a key role in advertising the Matchmaking Event. These programs helped to reach both UCSF faculty and potential applicants, fostering a welcoming atmosphere for the scholars once they arrived on campus.

A significant development coinciding with the Matchmaking Event was the launch of the PROPEL (Post-baccalaureate Research Opportunity to Promote Equity in Learning) program at UCSF. PROPEL aims to provide HU-background trainees with the research experience and career mentorship necessary to become competitive candidates for top-tier biomedical science PhD and MD/PhD programs (4). The possibility of participating in this program was a major factor in many of the applicants' (75%) decision to participate in the MME and, indeed, nearly all (27/31) of the trainees who made a match through the MME subsequently applied and joined PROPEL. In addition to institutional funds that serve a similar purpose, the PROPEL program successfully raised internal funds to support research mentor/trainee pairs, which provided additional incentives to faculty to hire trainees they met in the Matchmaking Event (5). However, participating in the Matchmaking Event does not automatically guarantee entry into PROPEL, as eligibility requirements for applying require participants to be hired into a full-time position at UCSF and wanting to preparing to apply to PhD and/or MD/PhD programs.

Additional Details of the Matchmaking Event

The first MME was organized by members of the UCSF Joining Underrepresented Minorities Students and Trainees with Investigators in Collaboration and Education (JUSTICE) group--which aims to promote diversity in the UCSF workforce--and occurred in January 2020. At the inaugural event, 26 mentors and 24 trainees participated in a two-hour in-person event

featuring "speed interviewing" to establish mentor-trainee relationships, followed by a social reception. Expanding on the success of the 2020 event, the January 2021 MME utilized pre-collected information about research opportunities and interests from mentors and trainees to pre-assign mentors with trainees of similar topical interests for one-on-one meetings. The event schedule allowed each trainee to have up to eight 15-minute mentor-trainee meetings, with the goal of meeting at least five potential mentors. In the week leading up to the event, trainees received their mentor-trainee pairing list, a guide on how to effectively communicate their educational training and research goals, and information on what to expect during the event. Mentors received their mentor-trainee pairing along with an informational guide on conducting the 15-minute mentor-trainee meetings. The informational guides provided to both mentors and trainees included sections on effective communication (see Supplemental Appendix 3 for guides). Mentors also received diversity, equity, and inclusion resources (see https://learning.ucsf.edu/content/diversity-equity-inclusion-resources for examples). They emphasized the importance of respecting and valuing different perspectives and experiences to create an inclusive and welcoming environment for all participants. Due to the COVID-19 pandemic, the 2021 MME was held virtually via Zoom to ensure accessibility for all participants. Technical support was provided to those who needed assistance navigating the virtual platform and accommodated various time zones to allow for broader participation across institutions.

Recent and current students were recruited from a wide range of universities:

California State Universities

- Bakersfield
- Chico
- Dominguez Hills
- East Bay
- Fresno
- Cal Poly Humboldt
- Long Beach
- Northridge
- Cal Poly Pomona
- Sacramento
- San Bernardino
- San Diego
- SFSU
- San Jose
- Cal Poly San Luis Obispo
- San Marcos

UC Schools:

- UC Berkeley
- UCLA
- UCSC
- UC Merced
- UC Riverside

Private schools:

- Pomona College
- Azusa Pacific University

- Biola University
- Dominican Univ.
- Harvey Mudd College
- Pitzer College
- University of La Verne
- USF

Other Schools:
- University of Florida

Misc:
- Emerson Collective
- 10,000 Degrees Foundation

References cited in Supplemental Appendix 1

1. UCSF. OIR UCSF. 2020 [cited 2022 Oct 18]. UCSF at a Glance. Available from: https://oir.ucsf.edu/ucsf-glance

2. U.S. Census Bureau. QuickFacts: United States [Internet]. 2021 [cited 2022 Oct 18]. Available from: https://www.census.gov/quickfacts/fact/table/US/PST045221

3. UCSF. UCSF Guides. 2022 [cited 2022 Dec 9]. UCSF Research Development Office (RDO): Grant Templates and Guides: NIH Diversity Supplements. Available from: https://guides.ucsf.edu/rdo/diversitysupplements

4. UCSF PROPEL. PROPEL. 2022 [cited 2022 Dec 9]. PROPEL: Post-baccalaureate Research Opportunity to Promote Equity in Learning. Available from: https://propel.ucsf.edu/propel-post-baccalaureate-research-opportunity-promote-equity-learning

5. Allen J, Abdiwahab E, Morris MD, Le Saux CJ, Betancur P, Ansel KM, et al. PROPEL: a scalable model for postbaccalaureate training to promote diversity in the biomedical workforce. J Microbiol Biol Educ. 2024 Sep 10;e0012224.

Supplemental Appendix 2. Details on the Matching Protocol Before the Event

Although this study focuses on data gathered for the 2021 Matchmaking Event, the inaugural event occurred in 2020. Since then, we have refined the interview matching mechanism. We want to highlight it here to help others recreate the event and use it to facilitate successful mentor-trainee relationships in their institutions. For the 2020 event, the mentors and trainees were randomly assigned into interview pairs. The feedback we received and our observations indicated that this could have been more efficient, so over the subsequent year, we developed a matching mechanism that prioritized matching based on the top five choices provided by both trainees and faculty. Therefore, building upon the 2020 event, pre-collected information about research opportunities and interests from mentors and trainees was used in the January 2021 MME to pre-assign mentors with trainees of similar topical interests for one-on-one meetings. The event schedule allowed each trainee up to eight 15-minute mentor-trainee meetings. The goal was for each trainee to meet with at least five potential mentors.

Student registrations were gathered using SmartSheets. SmartSheets allow you to create a form where the information is stored in a spreadsheet. We then give all faculty registrants access to this spreadsheet to review student information. SmartSheets was chosen because it allows trainees to attach a CV/resume, which gives faculty access to this database and provides the appropriate security to protect student information.

Faculty registration was done using Qualtrics. Faculty registrant information is downloaded to an Excel spreadsheet and sent to the students. Some faculty provide their lab website, and we provide a link to the UCSF faculty profiles page so students can learn more about each faculty registrant.

The registration forms for research mentors and trainees also provided an option to select one or more categories of research interest, such as basic science, outcomes/epidemiology, health disparities, clinical, or translational research. We used this information as a second level of priority to match people with similar interests after optimal pairing based on individual selections had been arranged. In addition, our new method adopted during the 2021 event provides an option for mentors to designate the times they are available, so they can still participate even if they are not available for the full three hours of the event.

Specific questions on student registration:

- Name/email/major
- Education stage
- Gradation date or anticipated graduation date
- Are you eligible for a diversity supplement (requirements listed)
- Are you a US citizen
- Describe how participation in an NIH diversity supplement will benefit your educational and career goals
- What research categories are you interested in (Basic Science Research, Outcomes/Epidemiology Research, Clinical/Translational Research, Community-Based Participatory Research, Health Disparities Research, Other)

- Primary, second, and third research areas of interest (Cancer Biology & Cell Signaling, Developmental & Stem Cell Biology, Human Genetics, Immunology, Neurobiology, Tissue / Organ Biology & Endocrinology, Vascular & Cardiac Biology, Virology & Microbial Pathogenesis, Computational Biology/Medical Informatics, Bioengineering, Epidemiology & Biostatistics, Reproductive Science, Chemistry and chemical biology, Biophysics, Biochemistry, Cell Biology, Other, No preference)
- Describe your research interests
- Prior research skills/experience (Quantitative analytical skills/experience, Qualitative analytical skills/experience, Experience working with human subjects or animals, Other)
- Describe in more detail your prior research-related experience that required data collection and analysis and the duration of the experience
- Demographics (gender, race/ethnicity, etc.)
- How did you hear about the event
- Attach a CV/Resume

Faculty registration questions:

- Name/email
- Please select the research category you are in (you can select multiple). ((Basic Science Research, Outcomes/Epidemiology Research, Clinical/Translational Research, Community-Based Participatory Research, Health Disparities Research, Other)
- First, second & third research areas of interest ((Cancer Biology & Cell Signaling, Developmental & Stem Cell Biology, Human Genetics, Immunology, Neurobiology, Tissue / Organ Biology & Endocrinology, Vascular & Cardiac Biology, Virology & Microbial Pathogenesis, Computational Biology/Medical Informatics, Bioengineering, Epidemiology & Biostatistics, Reproductive Science, Chemistry and chemical biology, Biophysics, Biochemistry, Cell Biology, Other, No preference)
- Description of your research
- Lab website
- What department are you in
- Demographics (gender, race/ethnicity, etc.)

Faculty & student "choices" survey:

- Name/email
- Five questions where they picked their 1st – 5th choice of people to interview/be interviewed by

NIH

Diversity Supplement Matchmaking Event

2021 Faculty Guide

Thank you for joining us for this year's NIH Diversity Supplement Matchmaking Event. We acknowledge that we are all living our lives in a different reality than what we probably imagined, and we want to applaud you for your continued commitment to students.

The UCSF School of Medicine Differences Matters initiative, the UCSF Office of Diversity and Outreach, the San Francisco State University SF BUILD program, and the UCSF Post-baccalaureate Research Opportunity to Promote Equity in Learning (PROPEL) continues our commitment to supporting trainees at the post-baccalaureate stage on their path to research-based Ph.D. programs. We are excited to provide you with the opportunity to engage with diverse students in this virtual setting.

The 2021 Matchmaking Event workgroup has put together this faculty guide to facilitate a smooth and impactful virtual experience for you. Please take some time to review the listed resources below to prepare you for the event from 9:00 am - 1:00 pm on Tuesday, January 19th, 2021.

Have questions on how to join a Zoom meeting? Please click on the blue link to learn how to join a meeting.

Joining a Zoom meeting

PROGRAM OUTLINE

Time	Program	Description
**9:00 - 9:30**	Welcome & Introductions	Join us in the main session space for a program welcome and a walkthrough of the event. Please join the main session using this zoom link
**9:30 - 9:45**	Break	
**9:45 - 10:45**	1st round of 4 interviews	Each interview will be 10 minutes long followed by a 5-minute break, you can use the 5-minute break to transition to your next interview.
**10:45 - 11:15**	Break	
**11:15 - 12:15**	2nd round of 4 interviews	Each interview will be 10 minutes long followed by a 5-minute break. You can use the 5-minute break to transition to your next interview.
**12:15 - 12:30**	Break	
**12:30 - 1:00**	**Q & A Session**<br>Use this zoom link to join the Q&A session	

NEED SUPPORT?

Return to the main session space and get your logistics questions answered!
If you need help and are not able to get back into the main room, email us at:
todd.nystul@ucsf.edu
yazmin.carrasco@ucsf.edu
jennifer.seuferer@ucsf.edu

Do you have questions about the virtual Speed Interviews of the NIH Diversity Supplement Matchmaking event? Here is a brief overview for your reference. Please continue reading below.

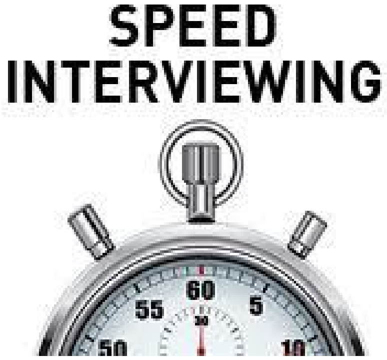

A Guide to Speed Interviews

This event is modeled on a “speed mentoring” event that matches applicants with UCSF faculty. Below is an adaptation of the handout given to UCSF faculty who attended the speed mentoring event.

What is Speed Interviewing?

Speed mentoring is a series of short, focused one-on-one conversations intended to serve as a way for you to get introduced to many applicants in a short period. After the 10-minute scheduled interview time has expired, a five-minute break follows. During this break, the applicant will exit your Zoom room. Please pay attention to your Zoom room waiting room during the 5-minute break as your next interviewee may have entered it.

What will I talk about with the applicants?

You may use your time with the applicant however you like, but it might be most efficient to start by giving the applicant a brief description of your research project and leave a minute or two at the end to ask them if they have any questions for you. This is an excellent opportunity to practice your elevator pitch. It is challenging to be concise when talking about your research and still form a cohesive story, so this guide introduces you to Randy Olson, a scientist turned filmmaker, and the AND, BUT, THEREFORE approach to storytelling. The AND, BUT, THEREFORE technique can be implemented to any story, including your research project, so when describing your research to the applicants, follow the following structure:

We know this….
AND we already observed this…
BUT we need to learn….
THEREFORE…. We tried something new.

If you would like to hear more about the AND, BUT, THEREFORE approach, you can look at his TED talk here.

What questions should you be ready to answer?

We have given the scholars the following ideas on questions to ask you:

- What will I be working on? (If the prospective research mentor doesn't share your role or desired contribution to their research project, this should be one of the first questions you ask)
- What qualities do you value most in a research trainee?
- How will you evaluate my progress?
- How often do you plan to meet with your mentee?
- Do you have regular group meetings? If so, how frequently? And what is the structure of those meetings?

Next Steps?

If you and the applicant spark each other's interest, please reach out to them directly after the Matchmaking Event. For Diversity, Equity and Inclusion Resources please visit the Graduate Faculty Development Program website here .

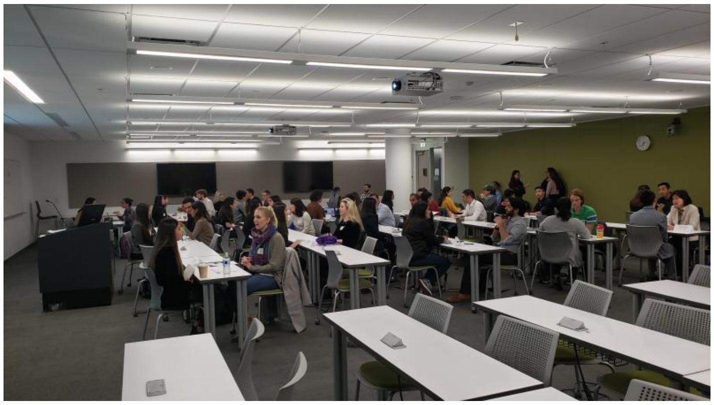

NIH Diversity Supplement Matchmaking Event 2020

NIH

Diversity Supplement Matchmaking Event

2021 Student Guide

Thank you for joining us for this year's NIH Diversity Supplement Matchmaking Event. We acknowledge that we are all living our lives in a different reality than what we probably imagined, and we want to applaud you in your continued commitment to learning.

The UCSF School of Medicine Differences Matters initiative, the UCSF Office of Diversity and Outreach (ODO), the San Francisco State University SF BUILD program, and the UCSF Post-baccalaureate Research Opportunity to Promote Equity in Learning (PROPEL) continues our commitment to supporting trainees at the post-baccalaureate stage on their path to research-based Ph.D. programs. We are excited to provide you with the opportunity to engage with UCSF faculty in this virtual setting.

The 2021 Matchmaking Event workgroup has put together this student guide to facilitate a smooth and impactful virtual experience for you. Please take some time to review the listed resources below to prepare you for the event from 9:00 am - 1:00 pm on Tuesday, January 19th, 2021.

Have questions on how to join a Zoom meeting? Please click on the blue link to learn how to join a meeting.

Joining a Zoom meeting

Differences Matter at UCSF | UCSF Office of Diversity and Outreach | SF BUILD | UCSF PROPEL

PROGRAM OUTLINE

Time	Program	Description
**9:00 - 9:30**	Welcome & Introductions	Join us in the main session space for a program welcome and a walkthrough of the event. Please join the main session using this zoom link
**9:30 - 9:45**	Break	
**9:45 - 10:45**	1st round of 4 interviews	Each interview will be 10 minutes long followed by a 5-minute break, you can use the 5-minute break to transition to your next interview.
**10:45 - 11:15**	Break	
**11:15 - 12:15**	2nd round of 4 interviews	Each interview will be 10 minutes long followed by a 5-minute break. You can use the 5-minute break to transition to your next interview.
**12:15 - 12:30**	Break	
**12:30 - 1:00**	**Q & A Session**	Use this zoom link to join the Q&A session

NEED SUPPORT?

Return to the main session space and get your logistics questions answered!
If you need help and are not able to get back into the main room, email us at:

todd.nystul@ucsf.edu
yazmin.carrasco@ucsf.edu
jennifer.seuferer@ucsf.edu

Do you have some butterflies in your stomach thinking about the Speed Interviewing portions of the NIH Diversity Supplement Matchmaking event? Here is a handy *Mentee's Guide to Speed Mentoring/Interviewing* for you to check out! Please continue reading below.

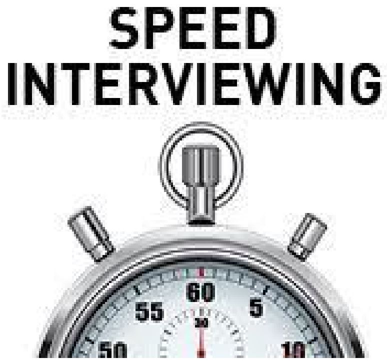

What is Speed Mentoring?

Speed mentoring is a series of short, focused conversations. You will virtually meet with several research mentors for 10-minute intervals each. After the 10-minutes have expired, a 5-minute break follows, allowing you to click the link for your next virtual interview and enter the virtual waiting room for the next scheduled faculty interview.

What is the purpose of speed mentoring?

The idea is for you to hear from multiple research mentors in different research areas. As you may know, the most effective mentoring relationships come from suitable mentor/mentee matches resulting from a natural affinity. Hence, we hope this initial interaction is a step in that direction.

What will I talk about with prospective UCSF research mentors?

Most of the 10-minutes are used by the mentors to share themselves and the research project, and how you will fit in that project. You want to state the following briefly:

- Name
- Background
    - Educational background (graduation date, major)
- Research interests/experiences
    - Share your research interests
    - Why are you interested in this research area?
    - Any research experiences that you have been involved in, if any.
- Career Aspirations / Goals
    - Share your career aspirations/goals
    - How will this training opportunity benefit your career aspirations/goals?

Can I ask questions?

Of course. The interview is a time for you to see if this is a possible research mentor you could work with and if

the research question they are pursuing is of interest to you, so ask away but do remember there is a 10-minute time limit. *There will be time at the end of the speed mentoring/session for more in-depth questions during the Q&A portion about the matchmaking process. Please refer to the Program Outline above.*

Some examples of questions you can ask are the following:

- What will I be working on? (If the mentor doesn't share your role or desired contribution to their research project, this should be one of the first questions you ask)
- What qualities do you value most in a research trainee?
- How will you evaluate my progress?
- How often do you plan to meet with your mentee?
- Do you have regular group meetings? If so, how frequently? And what is the structure of those meetings?

What will the UCSF research mentors talk to me about?

The questions below are potential questions that the research mentor may discuss.

- Name.
- Background.
- Lab composition.
- Motivation to participate in the matchmaking event.
- Summary of research project.
- Your role in the research project.

Next Steps?

After the Matchmaking Event, you are free to reach out to the mentors you are interested in working with. They, too, will be free to reach out to you. If you have any question, feel free to ask Yazmin Carrasco,PhD at yazmin.carrasco@ucsf.edu

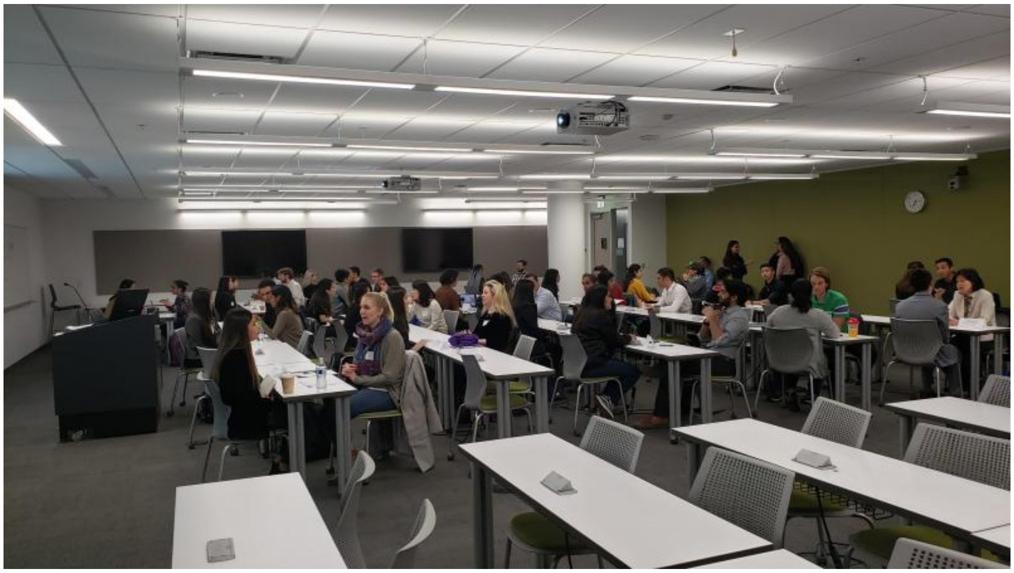

NIH Diversity Supplement Matchmaking Event 2020

Supplemental Appendix 4. Pre-Event Survey Measures on the Perceived Benefit of Diversity Supplement, Scientific Identity, Research Self-Efficacy, and Value of Diversity

In the pre-evet survey we collected data related to the perceived benefit of the diversity supplement, scientific identity, research self-efficacy, and the value of diversity among trainees and faculty to provide a more comprehensive understanding of participants' backgrounds and perspectives. These measures were included to enrich the interpretation of satisfaction and outcome data by considering key factors that influence the experiences and success of historically underrepresented (HU) students in STEMM fields. Previous research has highlighted the importance of these constructs in shaping students' intentions to pursue and persist in STEMM careers, particularly in interventions designed to increase the representation and academic achievement of HU students (1–3). By applying a social-ecological framework (4), we acknowledge the complex, intersecting levels—individual, environmental, and institutional—that influence the challenges HU students face in STEMM and aim to address these challenges through a holistic approach.

Measurement operationalization

The perceived benefit of applying/receiving an NIH diversity supplement was measured using selecting statements from a previously validated 17-item scale (1). Only items within the scope of the program's outcomes were chosen from the scale. The research team originally developed this scale to assess a program aimed at increasing the representation and academic achievement of HU undergraduates in the STEMM fields. Trainees were asked to determine "how useful" each item was to them: financial support, network and research experience, research career exploration, and belonging to a community of learners. The research team developed a 4-item scale to assess mentors' perceptions of the level of benefit of program involvement. Sample items include "opportunity to provide research mentorship for the trainee," "opportunity to have assistance moving my research forward," "enhance my mentoring portfolio for my CV," and "to inspire the next generation of scientists."

Science identity was measured among trainees by five items from the Scientific Identity Scale (5) that asked trainees to assess how much being a scientist is viewed as part of who they are. The scale was originally validated for undergraduate and graduate students. High reliability was reported in previous studies (6,7). The scale was reduced from 6 to 5 items deemed relevant to the educational attainment and experiences of the trainees. Sample items included "have a strong sense of belonging to the community of scientists," "feel like I belong in the field of science," and "have come to think of myself as a scientist."

Research self-efficacy was measured by nine items from the Scientific Self-Efficacy Scale (5), which assesses trainees' ability to function as scientists. Building on prior work, the scale included ten items that parallel the research experience for undergraduates, and these items were augmented with three more advanced items for graduate and postdoctoral fellows. The original scale was limited to items in the questionnaire that were appropriate for the trainees' training level. Trainees were asked "how confident" they felt performing each task, while mentors were asked to rank "how important" these items were. High reliability was reported in previous studies (6,7).

The perceived value of diversity was assessed using a 4- and 5-item scale, for mentors and trainees, respectively, using statements from the Cultural Competence Self-Assessment Questionnaire (8). However, items were modified by the research team to assess "how strongly

they agree" with each of the elements regarding the benefits of diversity in research teams. This measure was developed for the evaluation and was intended to assess participant perceptions of the diversity in STEMM and academic settings; as such, it serves as a measure of subjective program impact.

Descriptive statistics of the attendees

The perceived benefit of participating in the program was similar for both mentors and trainees. When asked to rank 4 statements about the perceived benefit of participating in the NIH Diversity Supplement program, the highest-ranked statement by mentors was 'being able to provide research mentorship for the trainee' (3.6 ± 0.6 SD), and trainees ranked research experience as 4.2 (SD 1.1) out of 5 (**Table 1**). Within the diversity assessment domain, responses were comparable between mentors and trainees. Both mentors and trainees agreed that research teams needed to reflect the broader population they study (4.8 ± 0.5 standard deviations [SD] for mentors, 4.7 ± 0.6 SD for trainees, p=0.26), and they both disagreed that having good grades is more important than hands-on experience (2.0 ± 0.8 SD for mentors, 2.1 ± 0.9 SD for trainees, p=0.42). Overall, trainees had a strong sense of science identity scoring ≥ 3 on a 4-point scale across all items, with the statement "I have a strong sense of belonging to the community of scientists" having the lowest score (3.2 ± 0.9 SD). Trainees on average had more experience in the following research self-efficacy areas related to: following instructions, generating answerable questions, analyzing data, creating explanations, interpretation, reviewing of literature, and reporting of results (p<0.05) compared to the preferred level expected of trainees reported by the mentors.

Table 1. Summary responses about diversity knowledge, skills, and beliefs collected using pre-session survey, by mentors and trainees (n=152)

	Mentors (n=73)	Trainees (n=79)	p-value
	Mean (SD)	Mean (SD)	
**Perceived benefit of participating in the NIH-diversity supplement program** [d]			n/a
Opportunity to provide research mentorship for the trainee [b]	3.6 (0.6)	--	
Opportunity to have assistance moving my research forward [b]	2.4 (0.6)	--	
Enhance my mentoring portfolio for my CV [b]	1.1 (0.3)	--	
To inspire the next generation of scientists [b]	2.9 (0.9)	--	
Financial support [a]	--	2.4 (1.2)	
Network experience [a]	--	2.9 (1.2)	
Research experience [a]	--	4.2 (1.1)	
Research career exploration [a]	--	3.0 (1.5)	
Belonging to a community of learners [a]	--	2.5 (1.4)	
**Diversity assessment** [a,c]			
Research teams need to reflect the broader population they study	4.8 (0.5)	4.7 (0.6)	p>0.05
Having good grades is more important than hands on experience	2.0 (0.8)	2.1 (0.9)	p>0.05
I understand that I'm a product of my upbringing and believe there are valid beliefs other than my own	--	4.6 (0.9)	--
I actively seek opportunities to connect with people who are different from me	--	4.6 (0.7)	--
People's experiences and background impact how they interact with me	--	4.3 (1.0)	--
There are many benefits that flow from a diverse scientific workforce	4.9 (0.2)	--	--
Research opportunities are not equally available to all due to systemic inequities	4.8 (0.6)	--	--
**Science identity** [b,e]			
I have a strong sense of belonging to the community of scientists	--	3.2 (0.9)	
I derive great personal satisfaction form working on a team that is doing important research	--	3.8 (0.5)	
I have come to think of myself as a 'scientist'	--	3.3 (0.8)	
I feel like I belong to the field of science	--	3.4 (0.8)	

Being a scientist is an important reflection of who I am	--	3.6 (0.5)	
**Research self-efficacy reported by trainee and preferred by faculty** [b,f]			
Can follow instructions	3.7 (0.5)	3.9 (0.3)	p=0.033
Knowing how to be a good team member	3.8 (0.5)	3.9 (0.3)	p=0.050
Able to generate answerable research questions	2.1 (0.8)	3.1 (0.8)	p<0.001
Able to analyze quantitative data	2.5 (0.8)	3.0 (0.8)	p<0.001
Create explanations for the results of the study	2.4 (0.7)	3.1 (0.8)	p<0.001
Able to interpret study or experiment results	2.6 (0.7)	3.1 (0.7)	p<0.001
Able to review scientific literature and/or reports to guide research	2.6 (0.8)	3.3 (0.7)	p<0.001
Report research results in an oral presentation or written	2.6 (0.8)	3.4 (0.8)	p<0.001
Identifying solutions to troubleshoot minor problems with research	2.9 (0.7)	3.1 (0.7)	p>0.05

Note: Data obtained by participants who completed the pre-session survey prior to the NIH event.
Abbreviations: NIH, National Institute of Health, DS, NIH diversity supplement, PROPEL, Post-baccalaureate Research Opportunity to Promote Equity in Learning, SFSU, San Francisco State University, UCSF, University of California San Francisco
a. Indicates item responses were along a 5-point Likert scale
b. Indicates item responses were along a 4-point Likert scale
c. Response values ranged from 1= strongly disagree to 5= strongly agree.
d. Response values ranged from 1=worst to 5=best.
e. Response values ranged from 1= strongly disagree to 4= strongly agree.
f. Response values ranged from 1= not confident at all to 4= very confident for trainees and 1=not at all important to 4=essential for faculty.

References cited in Supplemental Appendix 4:

1. Maton KI, Beason TS, Godsay S, Sto. Domingo MR, Bailey TC, Sun S, et al. Outcomes and Processes in the Meyerhoff Scholars Program: STEM PhD Completion, Sense of Community, Perceived Program Benefit, Science Identity, and Research Self-Efficacy. Marsteller P, editor. CBE—Life Sci Educ. 2016 Sep;15(3):ar48.

2. Camacho TC, Vasquez-Salgado Y, Chavira G, Boyns D, Appelrouth S, Saetermoe C, et al. Science Identity among Latinx Students in the Biomedical Sciences: The Role of a Critical Race Theory–Informed Undergraduate Research Experience. CBE—Life Sci Educ. 2021 Jun;20(2):ar23.

3. Stets JE, Brenner PS, Burke PJ, Serpe RT. The science identity and entering a science occupation. Soc Sci Res. 2017 May 1;64:1–14.

4. McLeroy KR, Bibeau D, Steckler A, Glanz K. An ecological perspective on health promotion programs. Health Educ Q. 1988;15(4):351–77.

5. Chemers MM, Zurbriggen EL, Syed M, Goza BK, Bearman S. The role of efficacy and identity in science career commitment among underrepresented minority students. J Soc Issues. 2011 Sep;67(3):469–91.

6. Estrada M, Woodcock A, Hernandez PR, Schultz PW. Toward a Model of Social Influence that Explains Minority Student Integration into the Scientific Community. J Educ Psychol. 2011 Feb 1;103(1):206–22.

7. Syed M, Zurbriggen EL, Chemers MM, Goza BK, Bearman S, Crosby FJ, et al. The Role of Self-Efficacy and Identity in Mediating the Effects of STEM Support Experiences. Anal Soc Issues Public Policy ASAP. 2019 Dec;19(1):7–49.

8. Mason JL. Cultural Competence Self-Assessment Questionnaire: A Manual for Users [Internet]. 1995 [cited 2022 May 25]. Available from: https://eric.ed.gov/?id=ED399684

Supplemental Table 1. Matchmaking Event Attendee Characteristics Among those who Made a Match, by Mentors and Trainees

Characteristic	Mentors who made a match (n=18)	Trainees who made a match (n=28)	p-value
	No. (%)	No. (%)	
Gender			p>0.05
Female	9 (50.0)	53 (53.6)	
Male	9 (50.0)	12 (42.9)	
Genderqueer or non-conforming	0 (0.0)	1 (3.6)	
Racial and ethnic group			p <0.001
White	11 (61.1)	3 (10.7)	
African American/ Black	0 (0.0)	5 (17.9)	
Asian	4 (22.2)	0 (0.0)	
Filipino, Hmong, Vietnamese	0 (0.0)	3 (10.7)	
Hispanic/Latinx	2 (11.1)	13 (46.4)	
Other	1 (5.6)	4 (14.3)	
Reported a disability	0 (0.0)	3 (10.7)	p>0.05

Supplemental Table 2. Matchmaking Event Attendee Characteristics of Trainees, by Whether they Made a Match

Characteristic	Trainees who didn't match (n=51) No. (%)	Trainees who made a match (n=28) No. (%)	p-value
Gender			p>0.05
Female	38 (74.5)	15 (53.6)	
Male	12 (23.5)	12 (42.9)	
Genderqueer or non-conforming	1 (2.0)	1 (3.6)	
Racial and ethnic group			p>0.05
White	4 (7.8)	3 (10.7)	
African American/ Black	7 (13.7)	5 (17.9)	
Asian	9 (17.7)	0 (0.0)	
Filipino, Hmong, Vietnamese	5 (9.8)	3 (10.7)	
Hispanic/Latinx	24 (47.1)	13 (46.4)	
Other	2 (3.9)	4 (10.7)	
Reported a disability	7 (13.7)	3 (10.7)	p>0.05
